# Savings and Losses of Scarce Virtual Water in the International Trade of Wheat, Maize, and Rice

**DOI:** 10.3390/ijerph19074119

**Published:** 2022-03-30

**Authors:** Hanfei Wu, Ruochen Jin, Ao Liu, Shiyun Jiang, Li Chai

**Affiliations:** 1 International College Beijing, China Agricultural University, Beijing 100083, China; hanfei.wu@ucdenver.edu (H.W.); ruochen.jin@ucdenver.edu (R.J.); 2018314060506@cau.edu.cn (A.L.); jasmine.jiang@okstate.edu (S.J.); 2 College of Economics and Management, China Agricultural University, Beijing 100083, China

**Keywords:** international cereal trade, virtual water trade, water scarcity, water savings and losses, virtual scarce water

## Abstract

The international cereal trade can mitigate global water stress by saving virtual scarce water (VSW). Based on bilateral trade data, this study assessed VSW savings and losses in the international trade of three major cereals (i.e., wheat, maize, and rice) from 2008 to 2017 by incorporating the water stress index (WSI) into a virtual water assessment. We found that the trade in wheat and maize saved a significant amount of VSW, while the rice trade led to increasingly severe losses of VSW. This study identified the top trades of VSW savings and losses for each cereal. Wheat and maize were primarily exported from the countries that are relatively abundant in water resources (e.g., United States, Brazil, Argentina, Russia) to water-scarce countries (e.g., Mexico and Egypt), whereas rice was exported mainly from India and Pakistan, two of the most water-stressed countries. We suggest that policy makers consider VSW savings and losses when making cereal trading decisions to alleviate global water stress.

## 1. Introduction

Global population growth is driving the demand for food, especially for wheat, rice, and maize, the three main cereal crops used for human consumption and animal feed. This requires more water because water is consumed in irrigation and food growth. For example, producing a kilogram of wheat requires 1827 L of water [1]. Water resources are limited and unevenly distributed worldwide. The rapidly increasing population growth and food requirements intensify water scarcity. By 2025, as the Food and Agriculture Organization of the United Nations has projected, two-thirds of the world population could face “stress” conditions, with between 500 and 1000 m^3^ of water per year per capita. However, the population is projected to reach ten billion by 2050 and global food demand is expected to rise accordingly. The contradiction between food demand and water shortages poses severe challenges to sustainable development and necessitates effective solutions.

At the same time, globalization is fueling the international trade of agricultural products. When a commodity is exported/imported, “virtual water” [2], the water embodied during agricultural production is implicitly exported/imported as well. About 20–24% of virtual water is traded internationally [3]. Virtual water is also known as the water that goes into the water footprint (WF). WF can be used as an indicator that reveals the lifecycle of water occupation in producing food. Depending on the water origin, it can be divided into three categories: green water, blue water, and gray water. Green water means the water stored in plants through evaporation, transpiration, and rainfall [4]. Blue water refers to surface runoff and ground water [5]. Grey water is the freshwater required to dilute polluted water [6,7].

Previous studies on virtual water have mainly quantified water transfers and the focus is on green water (rain water) and blue water (irrigation), with gray water rarely investigated. Liu et al. [8] concluded two groups. One is concerned with the amount of virtual water exchanged across borders between countries [4,9,10], across regions [11], within one country [12,13], and at a catchment level [14]. The other focuses on quantitative analyses of global water savings and losses associated with international and/or interregional trade [15,16,17,18]. However, these studies do not consider the environmental impacts of virtual water flows and how trade affects the sustainability of water use; the virtual water concept is limited in its usefulness in providing policy advice [19,20,21]. Moreover, there is not enough evidence to demonstrate that the saved water can mitigate global water stress [8].

Given that, water scarcity should be taken into account. The patterns of global trade in virtual water changes with the consideration of water scarcity [22,23]. Water scarcity is the ratio of total water withdrawal to total water availability, and the water stress index (WSI) is usually used to measure regional water scarcity. This index ranges from zero to one: the closer it is to one, the scarcer the local water resources, reflecting the severe water crisis [24]. The local water scarcity index divided by the global average water scarcity index is named the “water scarcity footprint” and can be used to calculate the indicator result of “water scarcity” [25]. Virtual scarce water trade refers to transfers of water embedded in goods between water-abundant countries/regions and water-scarce countries/regions. When the importer is a water-limited country and the exporter is a water-rich country, it can be considered sustainable, allowing a water-limited country to relocate water resources and alleviate water scarcity. Scarce water savings can be calculated according to the differences in water stress and water productivity between two regions. It can enhance the linkage between water savings and water scarcity, indicate where the saved water is more significant, and reveal “the hotspot regions of water savings and losses” [8]. Research on weighting the virtual water content by water scarcity focuses on regional water savings [26,27]. However, there are few studies concerning global scarce water savings.

Our study provides a comprehensive analysis of virtual water flows in global crop trade, including wheat, rice, and maize, from 2008–2017. We account for the sum of three water footprints, namely green water, blue water, and gray water, and quantify virtual scarce water savings and losses using a scarce water index as a weight. We also make a comparison of the changes over this decade. Our study reveals whether the global crop trade of wheat, rice, and maize are environmentally friendly or unsustainable, and identifies major virtual scarce waterflows. These results can provide advice for the development of relevant policies and tools to attenuate water scarcity, ensure the sustainability of water use, and meet sustainable development goals.

## 2. Methodology

### 2.1. Global Virtual Water Savings in Wheat, Maize, and Rice Trade

**Figure 1:**
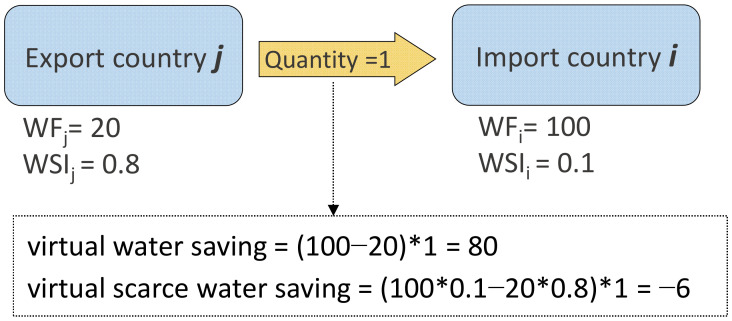
An example depicts how virtual water flow can save global water resources and virtual scarce water. WF refers to water footprint and WSI stands for water stress index.

Virtual water-saving occurs when the virtual water flows between two regions with different water productivities [28]. As depicted in Figure 1, when there is a difference in water productivity between two regions, i.e., producing a unit of crop requires 100 m^3^ of water in country i but only 20 m^3^ in country j, 80 m^3^ of water can be saved if country i imports a unit of crop from country j rather than producing this crop locally. Here, 80 m^3^ of saved water is defined as global virtual water saving (GVWS) in international crop-related trade [8].

The GVWS of cereal c (i.e., maize, wheat, and rice) can be calculated by Equation (1) below:

(1) $${\mathrm{GVWS}}_{c}^{\mathrm{ij}}=\;\left({\mathrm{WF}}_{c}^{i}-{\mathrm{WF}}_{c}^{j}\right)\;\times \;Q_{c}^{\mathrm{ij}}$$

where ${\mathrm{GVWS}}_{c}^{\mathrm{ij}}$ refers to the global virtual water saving in this trade (million m^3^/year); ${\mathrm{WF}}_{c}^{i}$ (${\mathrm{WF}}_{c}^{j}$) represents the virtual water footprint of cereal c required in the importing country i (exporting country j); $Q_{c}^{\mathrm{ij}}$ is the trade amount of cereal c between country i and j. ${\mathrm{GVWS}}_{c}^{\mathrm{ij}}$ with a positive value indicates that the trade c from i to j saves water, while a negative value refers to a water-losses trade.

Three components, namely green, blue, and grey water, were included in this study. Each cereal’s virtual water content in each country was obtained from Hoekstra’s database [29], which provides the water footprints of 352 crops and 106 animal products at the country level. This database has been widely used in previous studies [5,30,31]. The bilateral trade data were obtained from FAO’s database [32]. Due to data availability, this study covers 174 countries, accounting for 98.6% of the global population.

### 2.2. Virtual Scarce Water Savings in Global Wheat, Maize, and Rice Trade

Virtual water flow not only saves (losses) global water resources, but also impacts regional water scarcity [8]. For example, in Figure 1, the water footprint of the commodity in country j is 20, and 100 in country i. When the regions trade one quantity of this commodity, they save 80 virtual water, calculated by Formula (1) in Figure 1. When the WSI is taken into account, the scarce virtual water can be measured as the product of water footprint, trade quantity, and WSI. The numerical value is shown in Figure 1. For instance, in Figure 1, although the virtual water flow from j to i saves global water resources, it aggravates the regional water scarcity (losses 6 m^3^ of scarce water) because country j is more water-scarce than country i. The virtual scarce water can be estimated by employing the water stress index (WSI) [24]. WSI is a characteristic factor that can measure the regional water scarcity and has been widely used in previous studies to calculate the virtual scarce water [33]. The WSI of each country was obtained from the previous study [24].

Virtual scarce water saving (VSWS) can be estimated by using Equation (2):

(2) $${\mathrm{SVWS}}_{c}^{\mathrm{ij}}=\;\left({\mathrm{WF}}_{c}^{i}\times {\mathrm{WSI}}_{i}-{\mathrm{WF}}_{c}^{j}\times {\mathrm{WSI}}_{j}\right)\;\times \;Q_{c}^{\mathrm{ij}}$$

The product of ${\mathrm{WF}}_{c}^{i}$, ${\mathrm{WSI}}_{i}$, and $Q_{c}^{\mathrm{ij}}$ implies the scarce virtual water consumed by cereal c in region i, and the difference refers to the virtual scarce water savings generated by the trade. This study incorporated WSI into the water footprint itself and therefore is different from Lenzen et al. [22] and Vallino et al. [23].

The WSI of each country can be calculated by Equation (3):

(3) $$\mathrm{WSI}=\frac{1}{\left(1+e^{-6.4\mathrm{WTA}*\left(1/0.01-1\right)}\right)}$$

where WTA* refers to the modified water withdrawal-to-availability dimensionless indicator and was obtained from Pfister’s study [24]. According to Pfister et al. [24], a value of 0.5 for WSI is considered as the threshold between moderate and severe water stress.

## 3. Results

### 3.1. Impacts on Global Water Resources from 2008 to 2017

**Figure 2:**
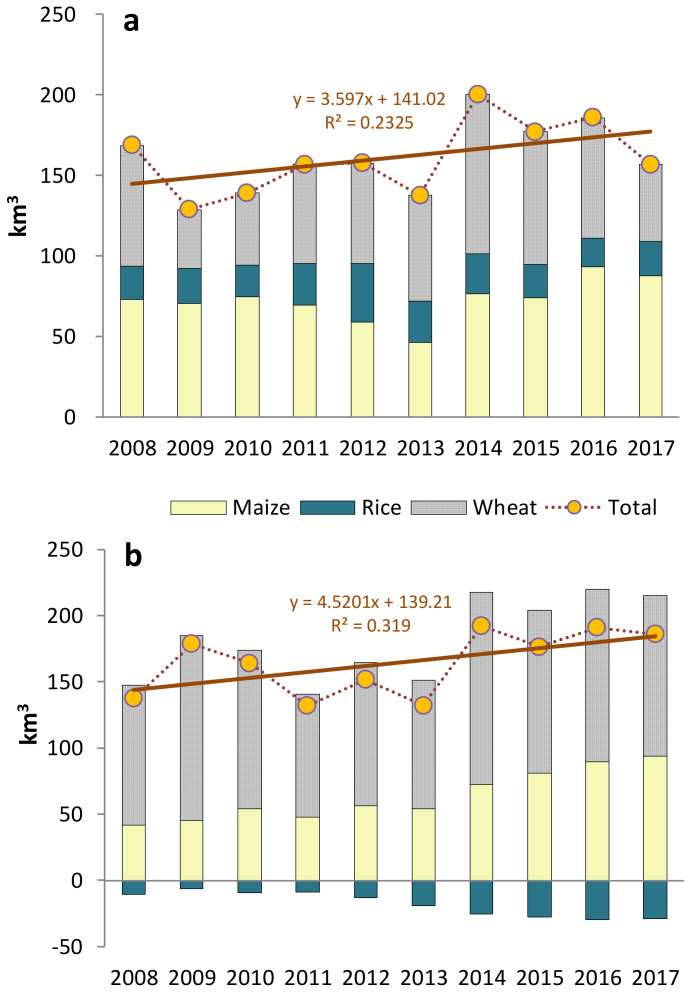
Net savings of (**a**) global virtual water and (**b**) global virtual scarce water in the international trade of maize, rice, and wheat from 2008 to 2017.

The figure below demonstrates the average global water savings and losses from 2008 to 2017. The ten-year average can eliminate outliers in an individual year and the impact of uncommon factors on trade. Figure 2a depicts the total global virtual water savings of maize, rice, and wheat in the decade from 2008 to 2017, with an average of 161 km^3^, peaking at 169 km^3^ in 2014 and bottoming at 129 km^3^ in 2009. The linear regression analysis can describe well the overall trend of the decade. From the analysis result, it suggests that the total amount of virtual water saved demonstrated a steadily and slowly rising trend. From 2008 to 2009, total virtual water savings fell sharply, reaching its lowest point in the decade in 2009, which can be largely attributed to the decline of this year’s wheat virtual water savings by 51.2% from the previous year. At the end of 2008, the global economy was severely hit by the economic crisis. Following the recession, the volume of the trade of food crops shrank in 2009, with US wheat exports falling by 0.4 million tonnes and European wheat imports decreasing by 0.46 million tonnes [34].

After 2009, total global virtual water savings increased gradually each year, except for a drop in 2013, mainly caused by the decrease of rice and maize virtual water savings. In 2012, as global rice yield rose, rice virtual water savings reached its peak in the decade, at 35.92 km^3^ (22.73% of the total saving in 2012). The total virtual water savings reduced to 137 km^3^ in 2013, with a trough of maize virtual water savings in the decade, at 46.13 km^3^ (33.67% of the total saving in 2013), resulting from a significant reduction of maize exports from the United States, the world’s largest producer and exporter of maize, by 23.32% from 31.53 million tonnes in 2012 to 24.1785 million tonnes in 2013 [35]. In 2014, the total virtual water savings surged to its highest point in the decade, when wheat virtual water savings also reached its peak in these ten years with 98.78 km^3^ (49.4% of the total saving in 2014), which was mainly due to an increase in production and trade volume of wheat in this year. Global wheat production stood at 723.38 million tonnes, an increase of 8.03 million tonnes compared to 2013, among which wheat production in Europe experienced the biggest growth, from 143.118 million tonnes in 2013 to 155.55 million tonnes in 2014 [36].

From 2015 to 2017, the total virtual water savings fluctuated between 157 km^3^ and 186 km^3^. In 2017, rice virtual water savings dropped significantly due to reduced wheat yield in two top wheat producing countries, the United States and Canada. From 2016 to 2017, wheat output in these two countries decreased from 62.83 million tonnes to 47.38 million tonnes, and from 32.14 million tonnes to 29.98 million tonnes, respectively [35].

A structure of agricultural trade that can save global virtual water is vital for the sustainable development of water resources. The biggest issue, however, is that water resources are unevenly distributed throughout the world, especially in water-scarce regions. Therefore, we should be concerned about whether the structure of global agricultural trade alleviates or aggravates the scarcity of global water resources. To address this problem, we introduced WSI to analyze the impact of the international trade of three major grains on global virtual scarce water resources.

Figure 2b presents virtual scarce water (VSW) savings from 2008 to 2017, with an average of 164 km^3^, peaking at 192 km^3^ in 2014, and bottoming at 132 km^3^ in 2011 and 2013. Similar to total virtual water savings, VSW savings demonstrated a steadily and slowly rising trend. Wheat VSW savings fluctuated with a peak at 144.89 km^3^ (75.46%) in 2014 and a trough at 92.7 km^3^ (70.22%) in 2011. Maize VSW savings exhibited an upward trend from 42.02 km^3^ (30.67%) in 2008 to 94.07 km^3^ (50.58%) in 2017, growing 123.9%. Different from wheat and maize VSW losses, rice VSW losses increased substantially, which did not affect the overall rising trend of global VSW savings, thanks to the more reasonable structure of maize trade that saved VSW significantly. It should be noted that the rice trade contributed to virtual water savings (as shown in Figure 2) but not VSW savings (as shown in Figure 2), and rice is the only grain that caused VSW losses. Comparably, wheat’s contribution to saving VSW is remarkable. Therefore, we need to focus more on VSW savings instead of merely paying attention to virtual water savings.

### 3.2. VSW Savings and Losses in Top Trades of Maize

**Figure 3:**
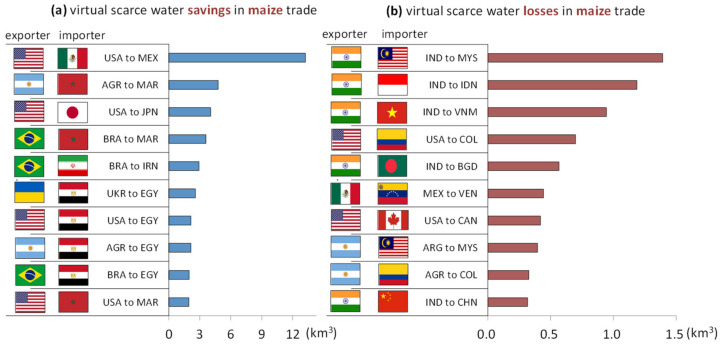
Top 10 maize trades of virtual scarce water (**a**) savings and (**b**) losses (the average during 2008 to 2017).

Figure 3a shows the top ten trade flows with the largest amount of VSW saved in global maize exports. Clearly, the largest VSW savings can be observed in maize exports from the United States to Mexico, at 13 km^3^, more than 2.5 times that of the second-ranked trade flow. The amount of VSW saved in the remaining nine trade flows ranges from 2 km^3^ to 5 km^3^. Overall, the United States, Brazil, and Argentina are the top three major exporters, having an advantage over other countries in the amount of VSW used for maize production, while Egypt and Morocco are the top two major importers. The United States, Argentina, and Brazil are the world’s important producers and exporters of agricultural products, which, combined, take up 73% of the world’s maize exports, occupying 42.2%, 16.9%, and 14%, respectively [37]. The annual maize production in the United States was basically stable at more than 10 billion bushels, and even reached 15.148 billion bushels in 2016. Maize’s planted area is 90 million acres with a ten-year average yield of nearly 160 bushels per acre. Iowa, Illinois, and Nebraska are the three top maize producing states in the United States, which are in the central region of the United States, with a suitable climate, fertile land, and a good drainage system [38]. The virtual water content (VWC) of maize in the United States is only 762 m^3^ per tonne, 40% below the global average, saving more VSW resources than most countries in the world. Moreover, water is relatively abundant in the United States and its WSI is only 0.499. Therefore, the United States owns a natural advantage in maize production, and maize exports from the U.S. are conducive to the sustainable development of the global water resources. Argentina is the smallest country of the three major exporters, but it overtakes the other two with the highest proportion of land used for agriculture. Argentina spans more than 30 degrees of latitude from north to south, so it has a diverse climate. The Pampas is a major producer of maize, and it is the most important region in the north, which is close to ports, making it easier to trade with other countries [37]. The VWC of maize in Argentina is 1139 m^3^ per tonne, also below the global average, but its greater advantage is that its WSI is only 0.352, which means that water resources are relatively rich in this country. These two figures suggest that maize exports from Argentina are beneficial to protecting water resources. Brazil’s unique climate and topography make it a good place to grow maize nationwide. Mato Grosso and Paraná, as the two largest maize producing areas in Brazil, are both located in the south of Brazil, with a typical subtropical climate and a warm and humid environment favorable for maize cultivation [37].

Based on the above analysis, we believe that countries, such as the United States, Brazil and Argentina, which consume less virtual water and have relatively abundant water resources, should be encouraged to undertake more maize production. For the future, in order to reduce the consumption of VSW worldwide, we need to enhance transactions between countries, lower import tariffs, and increase the transparency of maize trade policies and practices. We also recommend countries to build more proper and more durable trade partnerships.

Figure 3b describes the top ten trade flows with the largest amount of VSW lost in the global maize exports. The largest VSW losses can be observed in maize exports from Brazil to Malaysia, at 1.4 km^3^. India is the top exporter, occurring five times in the chart. Maize, which can grow all year round in India, is the most important crop of India besides rice and wheat. Maize acreage has been increasing because of the crop’s adaptability to different agro-climatic conditions and the low costs of labor involved. Andhra Pradesh and Karnataka are the two major producers of maize, accounting for 38% of the country’s maize production [39]. The rising maize output has driven up India’s maize exports, but this is not favorable if taking virtual water into account. In India, the VWC of maize is 2537 m^3^ per tonne, far above the global average of 1222 m^3^, meaning that maize production raises India’s water pressure, which contradicts the idea of sustainable development of water resources. In addition, India’s WSI is 0.967, indicating that it is a country with severe water scarcity. Thus, the Indian government should address the threat of maize exports to water resource sustainability and ponder whether it is reasonable to export large quantities of maize when its water resources are increasingly scarce.

### 3.3. VSW Savings and Losses in Top Trades of Rice

**Figure 4:**
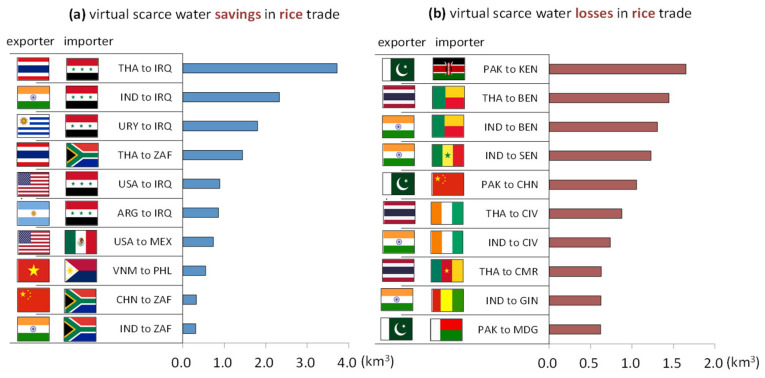
Top 10 rice trades of virtual scarce water (**a**) savings and (**b**) losses (the average during 2008 to 2017).

Figure 4a reveals the top ten trade flows with the largest amount of VSW saved in the global rice exports. The largest VSW savings can be observed in rice exports from Thailand to Iraq, at 3.7 km^3^, followed by that from India to Iraq, with 2.3 km^3^ and that from Uruguay to Iraq, with 1.8 km^3^. Overall, Iraq and South Africa are the top two major importers, appearing five times and three times in the chart, respectively. Iraq is facing the biggest environmental challenges, as 85% of renewable fresh water resources are used by the agricultural sector. Euphrates, located in the central and southern regions, is the major rice production area of Iraq. It has been in extreme drought conditions for a long time, with high temperature, low rainfall, and severe land desertification and soil salinization. Because of these adverse natural conditions, the country’s rice output cannot satisfy the demand for rice, making it one of the major importers of rice [40]. Hence, it is a sensible choice for Iraq to import rice, as the VWC of rice in Iraq is 12,055 m^3^ per tonne, far above the global average of 2414 m^3^, and its WSI is as high as 0.974. Thus, rice imports to Iraq are favorable to the sustainable development of water resources. South Africa is also a large importer of rice, and it is still unable to meet the huge demand caused by population growth and urban development, despite its annual rice output growth [41]. South Africa’s WSI is lower than Iraq’s, at 0.687, but it is still a country with severe water shortage. The amount of virtual water needed to produce rice in South Africa is nearly 2.5 times that of the global average; thus, importing rice to South Africa could alleviate the pressures on global water resources to some extent.

Figure 4b illustrates the top ten trade flows with the largest amount of VSW lost in the global rice exports. The top four largest VSW losses can be observed in rice exports from Pakistan to Kenya, at 1.6 km^3^, from Thailand to Benin, at 1.4 km^3^, from India to Benin, with 1.4 km^3^, and from India to Senegal, with 1.2 km^3^. Overall, India, Pakistan, and Thailand are three leading rice exporters, and 90% of the importers in the chart are African countries. The global average VWC of rice is 2414 m^3^ per tonne, making it one of the most water-intensive grains. India’s WSI is 0.967, thus it is among the countries with extremely scarce water resources. It also has the highest fresh water demand in the world, but 91% of its fresh water is used in the agricultural sector. Pakistan’s WSI is as high as 0.967, suggesting extreme water scarcity, with the per capita availability of water decreasing from 5600 m^3^ in 1947 to 1000 m^3^ in 2004. The underground water table in most parts of the country dropped by more than 7 m [42]. Thailand, a global rice exporter, faces the same problem, with a WSI of 0.534. By 2018, Thailand’s annual demand for fresh water was about 152 billion m^3^, with the agricultural water demand occupying 90.4%, at 114 billion m^3^ [43]. Rice cultivation accounts for almost 40% of Thailand’s total agricultural land area, and rice production in Thailand consumes the largest amount of water compared to other crops and other sectors [44]. Large rice production has eased the growing demand for food in India, Pakistan, and Thailand, but for the sustainability of water resources, governments in all these countries have to reconsider the rationality and sustainability of mass production and exports of rice.

### 3.4. VSW Savings and Losses in Top Trades of Wheat

**Figure 5:**
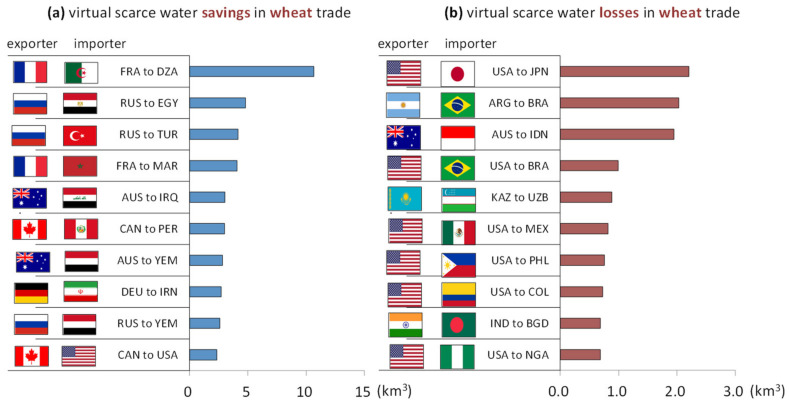
Top 10 wheat trades of virtual scarce water (**a**) savings and (**b**) losses (the average during 2008 to 2017).

Figure 5a indicates the top ten trade flows with the largest amount of VSW saved in the global wheat exports. Among them, wheat exports from France to Algeria save the largest amount of VSW, with 11 km^3^, 6 km^3^ larger than the exports from Russia to Egypt, the second largest contributor. France, Australia, and Canada all occur twice in the chart. Algeria experiences high water stress with its WSI at 0.79, and the VWC of wheat in this country is 3413 m^3^, almost 1.5 times that of Russia, which is the top exporter in the chart, appearing three times, and is the world’s largest exporter of wheat [38]. In Russia, the VWC of wheat is 2415 m^3^, over the global average of 1826 m^3^. However, Russia’s WSI is only 0.111, indicating it is rich in water resources. Therefore, production and exports of wheat from Russia can help alleviate the pressures of water resources in countries with a higher VWC of wheat or countries that face high water stress. In contrast to Russia, the VWC of wheat in France and Canada are below the global average, 587 m^3^ and 1542 m^3^ per tonne, respectively, so these two countries should be encouraged to partake in more wheat production.

Figure 5b demonstrates the top ten trade flows with the largest amount of VSW lost in the global wheat exports. The top three largest losses of VSW are in wheat exports from the United States to Japan, from Argentina to Brazil, and from Australia to Indonesia, with 2.2 km^3^, 2.0 km^3^, and 1.9 km^3^, respectively. The VWC of wheat in the United States is 2191 m^3^ per tonne, above the global average of 1826 m^3^ per tonne, which is higher than that in Japan, with 1242 m^3^ per tonne, mainly due to the low blue water consumption in Japan’s production of wheat, at only 5 m^3^ per tonne, far below the global average of 342 m^3^ per tonne. Consequently, there are virtual water losses in wheat exports from the United States to Japan. The United States is the top exporter, occurring fix times in the chart. United States’s WSI is 0.49, while Brazil’s is 0.06. The VWC of wheat in Mexico is 1076 m^3^ per tonne, less than that of the United States. Thus, it is not a preferable choice for the United States to export wheat to Brazil or to Mexico because it is a waste of water resources and there are higher costs when a less water-rich country exports agricultural products to a more water-rich country.

### 3.5. Limitations and Uncertainties

Van Vliet et al. [45] claimed that the sustainable management of water resources should not only consider water quantity but also quality. Their study demonstrates that global water scarcity is driven by both water quantity and quality. For some regions, in addition to exacerbating the water crisis by over-abstraction, sewage return flows can further pollute water sources, increasing water scarcity [46]. Moreover, Vallino et al. [47] claimed that except the physical water scarcity, economic water scarcity is also noteworthy. In some regions, the water is available but not usable because of infrastructural, economic, and social obstacles. Our study measured regional water scarcity using WSI, a quality-inclusive water scarcity index, which ignores water scarcity caused by the quality of available water. We encourage future studies using a more comprehensive index to measure the regional water scarcity when assessing virtual water trade.

There exists a certain amount of uncertainty in trade analysis. The international trade is affected by anti-globalization and trade conflicts, often reflected in tariffs and non-tariff barriers. When analyzed from an annual perspective, uncertainty often arises from outliers. We have used a ten-year average for analysis to avoid this effect. However, we advocate that future studies can reveal the virtual water trade pattern in a long-term time series and evaluate the trade from a time-series perspective.

## 4. Conclusions

This study examines the virtual water flows embodied in international trade in cereals (i.e., wheat, rice, and maize). From the results, global trade of the three major cereals has saved virtual water from 2008 to 2017. The overall trend in water savings is increasing. However, the water savings vary across the different cereal trades. For the maize trade, virtual water savings rose from 72.97 km^3^ in 2008 to 87.79 km^3^ in 2017. Although there was a significant decline in 2012 and 2013, the overall trend is still on the rise. Water savings for rice trade changed little over the decade, with a peak of 35.92 km^3^ in 2012. In contrast, though savings grew rapidly for the wheat trade from 2009 to 2014, they fell sharply after 2014, from 98.78 km^3^ to 47.95 km^3^.

The water stress index (WSI) has been introduced to assess global Virtual Scarce Water (VSW) savings and losses. Similar to virtual water, scarce virtual water savings also demonstrated an upward trend. From 2008 to 2017, while VSW savings in the wheat trade remained stable, VSW savings in the maize trade gradually escalated; contrastingly, VSW losses of rice trade experienced an ever greater increase from 10.49 km^3^ in 2008 to 28.98 km^3^ in 2017. Therefore, for water sustainability reasons, rice, a crop with a high virtual water content, should be produced in countries with a low WSI (i.e., water-rich countries) and then exported to water-scarce countries.

However, the rice trade was in the opposite way, where WSI reaches up to 0.96, India and Thailand were the two major exporter of rice during the studied period. Together with the results of the Lenzen et al. [22] study, we find that India, Pakistan, and Thailand are also the major exporters of scarce water. Thus, we encourage that these countries to re-examine their trade structure and pay more attention to the scarcity of water resources. For the trades of wheat and maize, the major exporters, including United States, Brazil, Argentina, and Russia, etc., are rich in water resources, resulting in significant VSW savings. Vallino et al. [23] studied the global trade in primary crops using the composite water scarcity index (CWSI), which is informed by physical and economic water scarcity. The application of the CWSI has transformed many countries from net exporters to net importers. They found that the current water flows between countries overlaps with the economic imbalances. Countries with lower CSWI received water from high CWSI countries, which account for as much as half of the total water flows. Therefore, the trade flows from India to Benin are not justified from either the WSI or CWSI perspective.

From the maize and wheat trade results, our study proves the benefit of the global cereal trade in saving VSW. We encourage policy makers to take water sustainability into account when making trade decisions. Meanwhile, water-rich countries are encouraged to enlarge their cereal exports within their capacity and take on more productive tasks without jeopardizing water security. Conversely, we recommend water-scarce countries to carefully assess the impact of cereal exports on their water resources before making trade decisions. What is more, when two countries establish trade links, the exporters should be a country with comparative advantage. When the opportunity cost of producing a water-intensive product is lower than that of its trading partners, a region is said to have a comparative advantage [48].

Although water scarcity is a regional problem, countries can achieve shared water sustainability and reduce local water scarcity by taking advantage of their comparative advantages, undertaking different production tasks and then trading globally. We therefore appeal to the intergovernmental cooperation in the cereal trade to jointly alleviate the global water crisis, as it is vital to build trust and stable partnerships between low-WSI and high-WSI countries.

## Data Availability

Not applicable.
